# Risk Factors for Postoperative Morbidity and Mortality after Small Bowel Surgery in Patients with Cirrhotic Liver Disease—A Retrospective Analysis of 76 Cases in a Tertiary Center

**DOI:** 10.3390/biology9110349

**Published:** 2020-10-22

**Authors:** Maximilian Wetterkamp, Cornelius J. van Beekum, Maria A. Willis, Tim R. Glowka, Steffen Manekeller, Rolf Fimmers, Michael Praktiknjo, Johannes Chang, Joerg C. Kalff, Tim O. Vilz

**Affiliations:** 1 Department of Surgery, University Hospital of Bonn, 53127 Bonn, Germany; max.wetterkamp@live.de (M.W.); cornelius.vanbeekum@ukbonn.de (C.J.v.B.); maria.willis@ukbonn.de (M.A.W.); tim.glowka@ukbonn.de (T.R.G.); steffen.manekeller@ukbonn.de (S.M.); kalff@uni-bonn.de (J.C.K.); 2 Institute of Medical Biometrics, Informatics and Epidemiology, Study Center Bonn, University Hospital of Bonn, 53127 Bonn, Germany; rolf.fimmers@ukbonn.de; 3 Department of Internal Medicine I, University Hospital of Bonn, 53127 Bonn, Germany; Michael.praktiknjo@ukbonn.de (M.P.); Johannes.chang@ukbonn.de (J.C.)

**Keywords:** small bowel surgery, liver cirrhosis, perioperative morbidity, perioperative mortality, risk factors

## Abstract

**Simple Summary:**

It is well known that the incidence of liver cirrhosis is increasing and it negatively affects outcome after surgery. While there are several studies investigating the influence of liver cirrhosis on colorectal, hepatobiliary, or hernia surgery, data about its impact on small bowel surgery are completely lacking. Therefore, a retrospective analysis over a period of 17 years was performed including 76 patients with liver cirrhosis and small bowel surgery. Postsurgical complications were analyzed, and 38 parameters as possible predictive factors for a worse outcome were investigated. We observed postsurgical complications in over 90% of the patients; in over 50%, the complications were classified as severe. When subdividing postoperative complications, bleeding, respiratory problems, wound healing disorders and anastomotic leakage, hydropic decompensation, and renal failure were most common. The most important predictive factors for those complications after uni- and multivariate analysis were portal hypertension, poor liver function, emergency or additional surgery, ascites, and high ASA score. We, therefore, recommend treatment of portal hypertension before small bowel surgery to avoid extension of the operation to other organs than the small bowel and in case of ascites to evaluate the creation of an anastomosis stoma instead of an unprotected anastomosis to prevent leakages.

**Abstract:**

(1) Purpose: As it is known, patients with liver cirrhosis (LC) undergoing colon surgery or hernia surgery have high perioperative morbidity and mortality. However, data about patients with LC undergoing small bowel surgery is lacking. This study aimed to analyze the morbidity and mortality of patients with LC after small bowel surgery in order to determine predictive risk factors for a poor outcome. (2) Methods: A retrospective analysis was performed of all patients undergoing small bowel surgery between January 2002 and July 2018 and identified 76 patients with LC. Postoperative complications were analyzed using the classification of Dindo/Clavien (D/C) and further subdivided (hemorrhage, pulmonary complication, wound healing disturbances, renal failure). A total of 38 possible predictive factors underwent univariate and multivariate analyses for different postoperative complications and in-hospital mortality. (3) Results: Postoperative complications [D/C grade ≥ II] occurred in 90.8% of patients and severe complications (D/C grade ≥ IIIB) in 53.9% of patients. Nine patients (11.8%) died during the postoperative course. Predictive factors for overall complications were “additional surgery” (OR 5.3) and “bowel anastomosis” (OR 5.6). For postoperative mortality, we identified the model of end-stage liver disease (MELD) score (OR 1.3) and portal hypertension (OR 5.8) as predictors. The most common complication was hemorrhage, followed by pulmonary complications, hydropic decompensation, renal failure, and wound healing disturbances. The most common risk factors for those complications were portal hypertension (PH), poor liver function, emergency or additional surgery, ascites, and high ASA score. (4) Conclusions: LC has a devastating influence on patients’ outcomes after small bowel resection. PH, poor liver function, high ASA score, and additional or emergency surgery as well as ascites were significant risk factors for worse outcomes. Therefore, PH should be treated before surgery whenever possible. Expansion of the operation should be avoided whenever possible and in case of at least moderate preoperative ascites, the creation of an anastomotic ostomy should be evaluated to prevent leakages.

## 1. Introduction

Liver cirrhosis (LC) is the common final stage of various chronic liver injuries such as viral or autoimmune hepatitis, alcoholic liver disease, and non-alcoholic steatohepatitis (NASH) caused by metabolic diseases or obesity [1,2,3].

The prevalence of LC has increased in the past decades while the life expectancy of patients suffering from LC improved since the introduction of novel therapies of viral hepatitis and optimized treatment strategies of comorbidities of cirrhotic liver disease. With a growing and aging collective of patients suffering from LC, the need for extrahepatic abdominal surgery in cirrhotic patients increases. Abdominal surgery like colorectal surgery or hernia repair yields higher perioperative morbidity and mortality in cirrhotic than in control groups, boosting cumulative costs of surgery in this clientele [4,5,6]. Complications of liver cirrhosis such as portal hypertension, ascites, malnutrition, renal dysfunction, or coagulopathy in addition to operative trauma and general anesthesia provoke various complications, resulting in a prolonged hospital stay and higher costs. The increased risk of mortality in patients with LC is the consequence of elevated bacterial infection rates, higher bleeding complications, and postoperative development of acute-on-chronic liver failure (ACLF) [7]. The surgical outcome depends on the severity of the underlying LC and the operative procedure [8,9]. While postoperative mortality for non-cirrhotic patients undergoing hernia repair, cholecystectomy, or bile duct exploration varies between 0.7% and 3.5%, it significantly increases in patients with LC to 8.3% in hernia repair and up to 25% in bile duct exploration, depending on the remaining liver function assessable as Child–Turcotte–Pugh Score (CTP) or the model of end-stage liver disease (MELD) Score [10,11,12,13,14,15]. To improve overall survival and outcome of patients with chronic liver disease, a strict surgical indication and a detailed examination of preoperative liver function need to be done.

Nevertheless, in Germany, there were about 800,000 small bowel or colorectal surgeries in 2018 according to a federal health report. Considering the prevalence of LC in the US 0.27% as a reference for the western population, there is a large number of patients with LC undergoing intestinal surgery [16]. There are a few retrospective analyses investigating the outcome of colorectal surgery in cirrhotic patients; however, there are no data available about morbidity and mortality of small bowel surgery in patients with LC, resulting in a lack of recommendation on how to minimize perioperative risk.

In this study, we retrospectively analyzed the intraoperative and postoperative course in patients with small bowel surgery and macroscopically or histologically confirmed liver cirrhosis. The aim of the study was to identify potential risk factors to optimize the patient’s condition prior to surgery and to be aware during the postoperative course in order to reduce postoperative morbidity and mortality.

## 2. Patients and Methods

The study was reviewed and approved by the Ethics Committee of the University Hospital of Bonn (Approval number 444/20).

### 2.1. Patient Selection

We retrospectively identified all patients who underwent resection of small bowel at the University Hospital of Bonn between 01/01/2002 and 07/31/2018 (*n* = 1781) using the German OPS Codes for small bowel surgery (5-450.0, 5-450.1, 5-454**, 5-460.0*, 5-460.1*, 5-461.5*, 5-465.0*/1*, 5-466.0*/1*, 5-467.0*/1*/3*/4*/5*). Only patients with the diagnosis of liver cirrhosis based on histological examination or intraoperative findings have been included in this study (*n* = 76). Data were obtained from the patient´s medical charts, physician letters, surgical reports, and anaesthesiologic protocols. Demographics and laboratory data, medical or interventional therapy, histological reports, the length of hospitalization, and duration of stay at an intensive care unit were analyzed.

### 2.2. Surgery

All patients underwent surgery of the small bowel such as resection with anastomosis or construction of a stoma and were treated with perioperative antibiotics (mezlocillin + metronidazole or ampicillin/sulbactam). Few patients received minor additional surgery (cholecystectomy, hernia repair, or liver excision ≥ one segment), which was also taken into account during analysis. All operations were performed by specialist surgeons at the University Hospital of Bonn.

### 2.3. Morbidity and Mortality

The Dindo/Clavien (D/C) score was used to classify postoperative complications [17]. Severe complications were defined as D/C grade ≥ IIIB. Morbidity was further categorized into:

- bleeding requiring transfusion of 2 or more units of red blood cells
- wound complications requiring vacuum-assisted closure (VAC) therapy or other surgical intervention
- anastomotic leakage and peritonitis
- redo procedures related to the initial small bowel surgery
- hydropic decompensation with drainage for more than ten days
- respiratory complications such as pneumonia requiring thoracentesis or mechanical ventilation
- hepatorenal syndrome or renal complications that necessitated renal replacement therapy (RRT)

Postoperative mortality was classified into 30-day and overall hospital mortality.

### 2.4. Statistical Analysis

Statistical analysis was performed using SPSS version 23 (IBM SPSS, Chicago, IL, USA). Continuous variables are expressed as mean (range) and discrete variables are reported as number (percentage).

We finally analyzed 38 possible predictive factors using uni-/multivariate analysis (Supplementary Tables S1 and S2). Logistic regression was used to analyze the occurrence of different types of complications. Multifactorial logistic regression models were derived by stepwise forward and backward selection to determine independent risk factors for postoperative complications and mortality. The resulting models were used to illustrate the predictive properties by plotting receiver operating characteristic curves (ROC curves) [18]. *p* values less than 0.05 were considered statistically significant.

## 3. Results

### 3.1. Patient’s Characteristics and Surgical Therapy

From 1781 patients between 01/01/2002 and 07/31/2018 who underwent small bowel surgery, 76 patients had histologically confirmed liver cirrhosis and were further analyzed (4.3%). For further information regarding the indication for surgery, see supplemental data: Table S3. The median age was 61.5 years with an interquartile range (IQR) of 15 years, 80.3% (*n* = 61) of patients were male. Laparotomy was performed in all cases, and an anastomosis was sutured in 77.6% (*n* = 59); all other patients (*n* = 17, 22.4%) underwent stoma creation. In 20 patients (26.3%) surgery was performed in an emergency setting.

In addition to small bowel surgery, 9.2% (*n* = 7) patients received cholecystectomy, and hernia repair was needed in 22.4% (*n* = 17) of all surgeries. Liver biopsy was performed in 14.5% (*n* = 11) patients, and 9.2% (*n* = 7) patients received liver surgery other than biopsy. 

The median postoperative length of stay for all patients was 23 days (IQR 26d), with a median stay of 2.5 days (IQR 7d) at an intensive care unit (Table 1).

Considering the etiology of cirrhosis, 27 patients (35.5%) suffered from alcoholic liver disease and 9 (11.8%) had viral hepatitis. In 36 patients (47.4%), no cause of the liver disease was found. Among the 76 patients, 52.6% (*n* = 40) were classified as CTP A, 39.5% (*n* = 30) as CTP B, and 7.9% (*n* = 6) as CTP C. MELD score was calculated with a mean score of 11.28 ± 4.68 with a range from 6 to 25. Analyzing the specific cirrhosis-related conditions before surgery, 21 patients (27.6%) with splenomegaly, 27 (35.5%) with portal hypertension, and 27 (35.5%) with varices were identified. Preoperative ascites was described as mild in 55.3% (*n* = 42), moderate in 30.3% (*n* = 23), and severe in 14.5% (*n* = 11) of patients. No patient suffered from hepatorenal syndrome preoperatively (Table 1).

Regarding severity of all comorbidities, 89.5% (*n* = 68) were classified as ASA III, 6.6% (*n* = 5) were accounted for ASA II, and 3.9% (*n* = 3) for ASA IV. Fifty patients (65.8%) suffered from prior cardiac (atrial fibrillation, coronary heart disease, chronic heart failure, or prior myocardial infarction), 27 (35.5%) renal (acute or chronic renal insufficiency), 20 (26.3%) neurological (prior stroke or epilepsy), and 28 (36.8%) metabolic (diabetes mellitus or obesity (Body Mass Index > 30 kg/m²)) conditions (Table 1).

### 3.2. Postoperative Complications

#### 3.2.1. General Postoperative Complications

Overall complications (D/C grade ≥ II) occurred in 90.8% of patients, whereas severe complications (D/C grade ≥ IIIB) were seen in 53.9% of patients (Table 2).

##### Univariate and Multivariate Analysis of General Postoperative Complications

**Figure 1:**
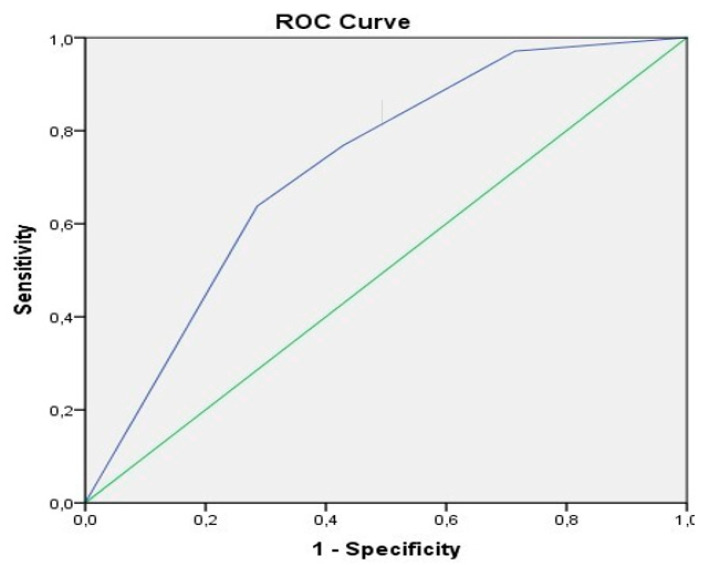
Receiver operating characteristic (ROC) curve complications Dindo/Clavien (D/C) grade ≥ II. Anastomosis 1st surgery and additional surgery combined, area under the curve (AUC) = 0.722.

Regarding overall complications with D/C grade ≥II in the univariate analysis, additional surgery and primary anastomosis were identified as significant (supplemental data: Table S1). In the multivariable analysis, we found primary anastomosis and additional surgery other than small bowel with an OR of 5.6 (primary anastomosis) and 5.3 (additional surgery) as predictors for increased postoperative complications (supplemental data: Table S2). The predictive property was illustrated by plotting receiver operating characteristic curves (ROC curve) for complications with D/C grade ≥II with an area under the curve (AUC) of 0.772 (Figure 1).

For severe postoperative complications (D/C grade ≥ IIIB), we observed pre-existing neurological conditions and high ASA score as risk factors in univariate analysis (supplemental data: Table S1). Multivariate analysis showed pre-existing neurological conditions and thrombocytopenia as predictors for elevated severe postoperative complications with an OR of 5.6 (pre-existing neurological conditions) and an OR of 5.0 for thrombocytopenia (supplemental data: Table S2).

#### 3.2.2. Bleeding Complications

The most common postoperative complication was hemorrhage (*n* = 31, 40.8%) requiring transfusion of at least 2 red blood cell units (Table 2).

##### Univariate and Multivariate Analysis

We analyzed 38 factors and could demonstrate in a univariate analysis that male sex was a risk factor (*p* < 0.001) for bleeding requiring transfusion (supplemental data: Table S1**)**. In our multivariate analysis, we found an OR of 17.8 (male sex), an OR of 4.1 (portal hypertension), and an OR of 4.7 (emergency procedures). Interestingly, postoperative bleeding complications were not associated with coagulation parameters or stage of liver disease (supplemental data: Table S2).

#### 3.2.3. Respiratory Complications

Respiratory complications after small bowel surgery were the second most common adverse event (38.2%, *n* = 29). From those patients, 21 (27.6%) suffered from pneumonia, 17 patients (22.4%) had recurrent pleural effusion requiring thoracocentesis, and twelve patients (15.8%) needed intubation and mechanical ventilation (Table 2).

##### Univariate and Multivariate Analysis

Regarding postoperative respiratory complications, leucocytes deviating from their normal range, low number of platelets, diagnosis of hepatocellular carcinoma, high ASA score, and emergency surgery were the most important risk factors (supplemental data: Table S1). The multivariable analysis determined an OR of 10.1 for a low number of platelets, an OR of 8.8 for additional surgery, and an OR of 2.1 for leucocytosis/-penia (supplemental data: Table S2).

Pneumonia: Regarding postoperative pneumonia, a low number of platelets, moderate ascites, hepatocellular carcinoma, pre-existing neurological conditions, and high ASA score were significant predictors in univariate analysis. We identified a low number of platelets, additional surgery, and leucocytes deviating from their normal range in multivariate analysis as risk factors.

Pleural effusion: We identified hepatocellular carcinoma, ASA, and encephalopathy as risk factors for pleural effusion requiring thoracocentesis in univariate and hepatocellular carcinoma and ASA in multivariate analysis.

Re-intubation and mechanical ventilation: ASA score, pre-existing cardiac and neurological conditions were significant predictors in univariate analysis. The multivariate analysis determined only ASA classification as significant.

#### 3.2.4. Wound Healing Disorders

Wound healing disturbances defined as wound revision requiring general anesthesia like wound debridement or VAC therapy could be observed in 32.9% of patients (*n* = 25) (Table 2).

##### Univariate and Multivariate Analysis

Only moderate ascites was significant (*p* = 0.049) in univariate analysis (supplemental data: Table S1). The multifactorial analysis for wound healing disorders identified no specific risk factor (supplemental data: Table S2). 

#### 3.2.5. Hydropic Decompensation

Hydropic decompensation was defined as intraoperative inserted drainages required for >10 days or postoperative abdominal paracentesis and could be observed in 30.3% of patients (*n* = 21) (Table 2).

##### Univariate and Multivariate Analysis

We identified 11 different risk factors for hydropic decompensation (CTP score, leucocytes differing from their normal account, low platelets, portal hypertension, esophageal varices, and additional surgery) as significant in univariate analysis (supplemental data: Table S1). In the multivariable analysis, we found CTP classification with an OR of 3.7 (the risk of hydropic decompensation of a patient with CTP group B is 3.7 times higher compared to CTP group A) and portal hypertension with an OR of 3.4 (supplemental data: Table S2).

#### 3.2.6. Redo Procedures

Reoperations were necessary in 21 patients (27.6%), the patients needed at least 1 and up to 18 redo procedures (Table 2). The median was three redo procedures.

##### Univariate and Multivariate Analysis

ASA classification was the only predictor for redo procedures related to the initial small bowel surgery identified in univariate analysis (supplemental data: Table S1). The OR calculated in multivariate analysis was 7.7 (supplemental data: Table S2). With each elevation of the ASA classification, the risk for redo procedures increases by 7.7 times in this study.

#### 3.2.7. Renal Replacement

Postoperative hemodialysis or hemofiltration due to renal failure was necessary in 11 cases (14.5%) (Table 2).

##### Univariate and Multivariate Analysis

**Figure 2:**
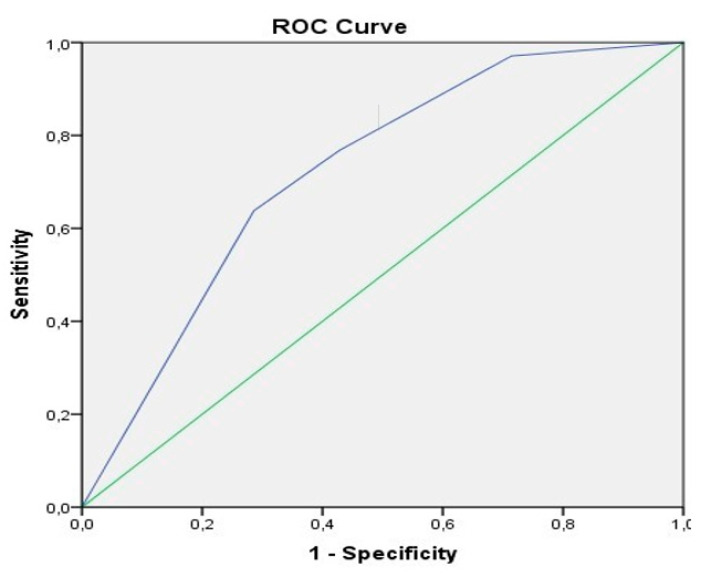
ROC curve renal replacement therapy. Thrombocytopenia < 100 (G/L) combined with ascites group and ASA classification, AUC = 0.878.

The univariate analysis defined 13 different risk factors including MELD score, portal hypertension, encephalopathy, emergency surgery, and primary anastomosis (supplemental data: Table S1). The multivariable analysis determined a low number of platelets (OR 13.2), ascites group (OR 2.7), and the ASA classification (OR 14.9) as significant predictors with an AUC of 0.878 (supplemental data: Table S2). With each elevation of the ASA classification, the risk of postoperative renal replacement therapy increases by 14.9 times in this study. The ROC curve is shown in Figure 2.

#### 3.2.8. Anastomotic Leakage

Anastomotic leakage occurred in 11.8% of patients after small bowel surgery (*n* = 9) (Table 2).

##### Univariate and Multivariate Analysis

Interestingly, mild and moderate ascites were the only significant risk factors for anastomotic leakage found in univariate analysis (supplemental data: Table S1). The multivariate analysis determined an OR of 11.9 for moderate ascites as a predictor for anastomotic leakage (supplemental data: Table S2).

#### 3.2.9. Hospital Mortality and 30-day Mortality

No patient died during the procedure, whereas 9 patients (11.8%) died during the postoperative stay. Three patients deceased within the first 30 days after surgery, all due to abdominal sepsis (Table 3).

##### Univariate and Multivariate Analysis

**Figure 3:**
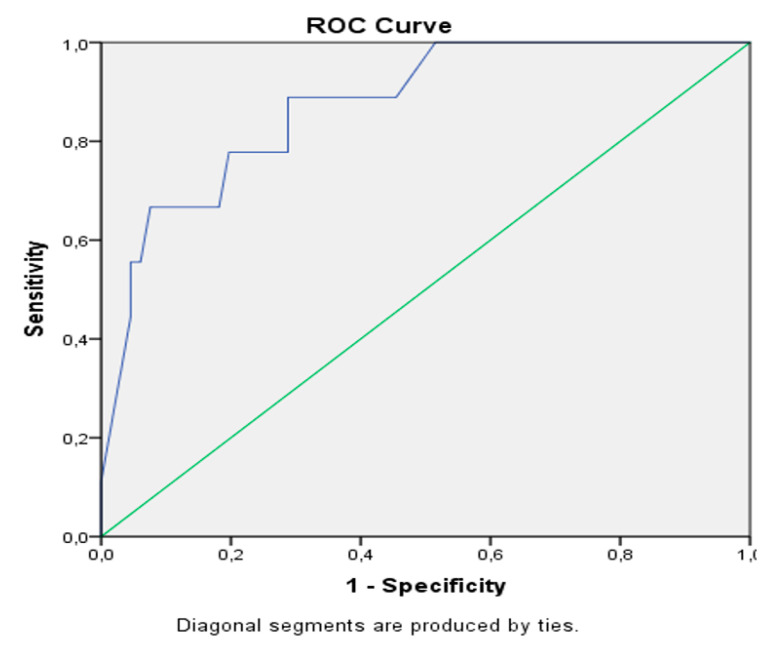
ROC curve mortality. MELD score combined with portal hypotension, AUC = 0.873.

From the investigated 38 possible predictive factors, we identified 17 different risk factors for hospital mortality in the univariate analyses. The most important factors were impaired renal function (elevated creatinine), limited coagulation, advanced stage of liver disease (elevated MELD and CTP score), portal hypertension, high ASA Score, and emergency surgery (supplemental data: Table S1). However, only MELD score and portal hypertension were significant predictors for a fatal outcome in multivariable analysis with an odds ratio (OR) of 1.3 (MELD) and 5.8 (portal hypertension) and an AUC of 0.873 (supplemental data: Table S2). The ROC curve is illustrated in Figure 3. With each additional point of the MELD score, the risk of postoperative mortality increases by 1.3 times in this study.

## 4. Discussion

In western countries, LC is increasing due to NASH. Generally, LC is a well-known cause of perioperative morbidity and mortality following abdominal surgery and has not been reported to decrease in the past decades [10,13,19]. In particular, the perioperative risk factors for hepatobiliary, colorectal, and hernia surgery are well established, showing that increasing CTP, CTP in combination with ASA score, and an increasing MELD score may act as a predictive factor for a worsened surgical outcome [6,16,20,21]. However, there are no data about the perioperative risk of patients with liver cirrhosis undergoing small bowel surgery.

In this study, we analyzed morbidity and mortality in 76 cirrhotic patients with elective or emergent surgery of the small bowel, using not only the CTP and MELD score but also examined the influence of 38 different variables in univariate and multivariate analyses. For all observed complications during the postoperative course, we could identify independent risk factors that could predict a worse outcome.

First, we demonstrated the devastating influence of LC on the postoperative outcome with surgical complications D/C grade ≥ II in over 90% and severe complications (≥IIIB) in 54% of the patients. Compared to cirrhotic patients undergoing large bowel surgery, the outcome is worse in our cohort [15,22,23,24,25]. At first glance, this is very surprising because small bowel resection is usually associated with lower adverse events than colonic surgery. However, when comparing the condition of our study with other trials, we had a longer operation time, a higher ASA score, and a higher CTP score [23]. In the multivariate analysis, we found “additional surgery other than small bowel resection” and “creation of a bowel anastomosis” as risk factors for overall complications and “preexisting neurological disorders” and “thrombocytopenia” as risk factors for severe complications. Whereas neurological disorders and thrombocytopenia (as a result of worse liver function and portal hypertension) are difficult to improve, “additional surgery” like cholecystectomy or hernia repair should be avoided during small bowel resection whenever possible. The decision on how to proceed in case of necessary small bowel resection is difficult. The risk of a worsened outcome after anastomosis creation is increased fivefold. Furthermore, we observed a leakage rate of 12%, which is comparable to rectal surgery. However, small bowel ostomy is associated with high morbidity (for example, fluid loss) too, especially in patients with renal impairment. Furthermore, when there is less than 150–200 cm small bowel orally of the ostomy, there is a threat of short bowel syndrome, which will be a major problem in mostly malnourished cirrhotic patients. A potential decision-making tool is the number of ascites, as we could demonstrate in our work that the risk of anastomotic leakage is increased eleven times when ascites is present. Therefore, we recommend decision-making “pro ostomy” (for example, anastomotic stoma to increase the chance of a stoma relocation) in patients with more than 200 cm and the presence of ascites. Nevertheless, the surgeon and the patient should be aware that there is a high probability that the ostomy will last forever.

When further analyzing the morbidity in our cohort, the most common complication in over 40% of all patients was hemorrhage. This is comparable to colorectal procedures and LC as shown by Lee et al. [26]. In our multivariate analysis, we identified several risk factors. The relation between portal hypertension with its collaterals and a higher blood loss is reasonable. The second risk factor, emergency surgery, can be explained by the lack of time to substitute plasmatic clotting factors and thrombocytes in an urgent setting. Furthermore, all patients in our cohort underwent open surgery, which is associated with an increased blood loss even in patients without impaired coagulation. As Kim et al. and Zhou et al. could demonstrate in their studies, laparoscopy is safe and feasible even in cirrhotic patients and should, therefore, be performed whenever possible [27,28].

The second most common complication in our cohort was respiratory decompensation (38%). This is a little higher than as described by Nguyen et al. [15]. However, in the studies from Hübner et al. and Jurt et al. analyzing patients undergoing colon surgery without liver cirrhosis, the respiratory failure rate was six times lower [29,30]. In a multivariate analysis, we found “additional surgery”, “low platelets”, and “abnormal leucocytes before surgery” as risk factors for developing pneumonia and “ASA score” for developing pleural effusion and the need for reintubation in the postoperative course. Other studies identified the ASA score as a risk factor as well [30]. With the exception of additional surgery, these are no modifiable parameters. Therefore, the perioperative treatment in patients with thrombocytopenia and higher ASA score should include goal-directed volume treatment and fluid balancing to avoid an overload, early mobilization, and breathing exercises as pneumonia prophylaxis as well as sonographic controls of the pleural cavity for drainable effusion [29,30]. Furthermore, prophylactic gastric tubes as a risk factor for aspiration should be avoided.

Another common complication after surgery in patients with LC is hydropic decompensation during the postoperative course. We found CTP score as well as portal hypertension as independent risk factors. In these patients, we strongly recommend to place a temporary ascites drain in order to avoid wound healing disturbances or hernia. Especially in patients with CTP C cirrhosis (7.4-fold risk) or CTP B combined with portal hypertension (7.1-fold risk), the implantation of a permanent drain (for example, PleurEx^©^ catheter) to avoid peritonitis due to long-lasting temporary drain should be carefully evaluated [31,32].

In our cohort, we observed hepatorenal syndrome with the need for dialysis in 14.5% of all cases. This is significantly higher as described by Ziser et al. or Del Olmo et al. [8,10]. However, in these manuscripts, all kinds of surgical procedures were included and almost exclusively patients with CTP A. In our study, independent risk factors for renal replacement were a low number of platelets before surgery, age, and especially ASA score with an increase of 15 times per ASA point (for example, ASA 3 results in a 30 times increased risk!). This correlates to the study from Ramonell, who could also identify ASA score as well as hypoalbuminemia and age as risk factors for renal failure [33]. Because age and ASA score cannot be improved, particular attention should be paid to renal function during and after surgery with thoughtful fluid therapy, an early substitution of albumin, and the start of terlipressin treatment. 

Wound healing disorders (31%) and the need for redo procedures (27%) were common complications in our cohort. This is no surprise since patients with liver cirrhosis often suffer from malnutrition, which is a well-known risk factor for surgical complications [34,35]. Interestingly, in contrast to other studies, decreased albumin was not associated with a worse outcome [36,37,38]. However, increasing ASA classification increased the risk by 7.7 times.

Finally, we analyzed the mortality of small bowel resection in cirrhotic patients. Twelve percent of the patients died (D/C grade V) during the postoperative course, most of them due to abdominal sepsis. This is striking because small bowel resection is known to be a surgical procedure with very low morbidity and mortality. As risk factors, we could identify the MELD score and again portal hypertension. The significant higher mortality in patients with portal hypertension has been described in other trials before [15]. Those patients are more likely to suffer from ascites, encephalopathy, anemia, and bleeding [39,40]. We tried to identify a cut-off MELD score (MELD > 9, >14, >17) as described for colorectal surgery and non-hepatic surgery in order to have an easy predictive value [20,23]. In our univariate analysis, all cut-offs were significant, but the MELD score itself was superior in univariate as well as in multivariate analyses. However, we could demonstrate that with each additional point of the MELD score, the risk of postoperative mortality increases by 1.3 times. In contrast to other studies, neither the ASA score nor the CTP score was predictive factors.

Although we were able to demonstrate the catastrophic influence of liver cirrhosis on the outcome after small bowel operations for the first time, there are various limitations of our study. The retrospective design of a single-center study and the small cohort of patients with liver cirrhosis undergoing small bowel surgery may overpower the influence of the found predictors. Furthermore, this study includes only open surgical procedures, which are known to have a higher blood loss in a cohort without cirrhosis and may have a higher incidence of ascites and a higher rate of postoperative liver failure compared to minimally invasive procedures in patients with cirrhosis [41,42].

## 5. Conclusions

For the first time, we could demonstrate the devastating influence of small bowel resection in patients with liver cirrhosis. The most common risk factors for postoperative morbidity and mortality in the multivariate analysis were additional surgery, poor liver function, portal hypertension (with its consequences of ascites and thrombocytopenia), and high ASA score. Therefore, in elective surgery, the indication should be made strictly and whenever possible only after the treatment of portal hypertension. During surgery, escalation of the operation, for example, cholecystectomy in case of gall stones or asymptomatic hernia repair, should be avoided. In the case of preoperative ascites and other well-known risk factors for anastomotic leakages (for example, sarcopenia, immunosuppression), the creation of an anastomotic ostomy should be considered. 

## Figures and Tables

**Table 1:** Baseline characteristics of the study population.

Factors	Total (*n* = 76)/Median (IQR)
Sex	
Male	61 (80.3%)
Female	15 (19.7%)
Age (years)	61.5 (15)
Age group	
<40 years	1 (1.3%)
41–74 years	66 (86.8%)
>74 years	9 (11.8%)
Creatinine (mg/dL)	1.1 (0.79)
Bilirubin (mg/dL)	0.65 (0.81)
PT (INR)	1.1 (0.2)
Thrombocytes (G/L)	185 (129)
Leucocytes (G/L)	7.03 (4.94)
MELD score	10 (7)
CTP classification	
A	40 (52.6%)
B	30 (39.5%)
C	6 (7.9%)
ASA classification	
II	5 (6.6%)
III	68 (89.5%)
IV	3 (3.9%)
Etiology of liver cirrhosis	
Alcoholic	27 (35.5%)
HBV and/or HCV	9 (11.8%)
Cryptogenic	36 (47.4%)
PBC or PSC	4 (5.2%)
Splenomegaly	21 (27.6%)
Portal hypertension	27 (35.5%)
Varices	27 (35.5%)
Ascites	
No/mild	42 (55.3%)
Moderate	23 (30.3%)
Severe/refractory	11 (14.5%)
Encephalopathy	
No	70 (92.1%)
Grade 1/2	5 (6.6%)
Grade 3/4	1 (1.3%)
Presence of HCC	4 (5.3%)
Pre-existing metabolic condition/diabetes	28 (36.8%)
Pre-existing cardiac condition	50 (65.8%)
Pre-existing renal condition	27 (35.5%)
Pre-existing neurological condition	20 (26.3%)
Pre-existing respiratory condition	14 (18.4%)
Surgery location	
Duodenum	14 (18.4%)
Jejunum	39 (51.3%)
Ileum	37 (48.7%)
Incision-suture time (min)	209.50 (153)
Elective vs. emergency surgery	56 (73.7%) vs. 20 (26.3%)
Anastomosis vs. ostomy	59 (77.6%) vs. 17 (22.4%)
Postoperative hospital days	23 (26)
Postoperative hospital days at ICU	2.5 (7)

IQR: interquartile range, PT: prothrombin time, INR: international normalized ratio, HBV: hepatitis B virus, PBC: primary biliary cholangitis, HCV: hepatitis C virus, PSC: primary sclerosing cholangitis, HCC: hepatocellular carcinoma, ICU: intensive care unit.

**Table 2:** Morbidity and mortality.

Complication	Total (*n* = 76)
Complications Dindo-Clavien ≥ II	69 (90.8%)
Severe complications Dindo-Clavien ≥ IIIB	41 (53.9%)
Bleeding requiring transfusion	31 (40.8%)
Respiratory complication (pneumonia, thoracentesis, mechanical ventilation)	29 (38.2%)
Wound healing disorder	25 (32.9%)
Hydropic decompensation	23 (30.3%)
Redo procedures	21 (27.6%)
Renal complication (renal replacement therapy)	11 (14.5%)
Anastomotic leakage	9 (11.8%)
Hospital Mortality	9 (11.8%)
30-day mortality	3 (3.9%)

**Table 3:** Mortality cases.

Sex	Age	CTP	MELD	Operation	Complications	Cause of Death	Day
M	69	A	10	Jejunal and ileal anastomosis	Bleeding, Resp, Renal, AnaLeak, Redo (2)	Sepsis (peritonitis)	5
F	72	B	15	Jejunal anastomosis	Bleeding, Resp, Renal, WHD, HRS, HyDecomp, Redo (1)	Liver failure (limited therapy)	148
M	60	A	12	Ileal anastomosis	Peritonitis, Redo (3)	Sepsis (peritonitis)	3
M	67	B	20	Ileal anastomosis	Resp, Renal, HRS, HyDecomp	Sepsis (pneumonia, limited therapy)	36
M	54	C	18	Jejunal anastomosis	Resp, Renal, HRS,	Sepsis (pneumonia)	31
F	72	C	16	Jejunal anastomosis	Bleeding, Resp, Peritonitis	Sepsis (peritonitis)	2
M	60	B	25	Ileal stoma	Resp, Renal, WHD, HyDecomp, Peritonitis, Redo (1)	Hemorrhagic shock	40
M	69	A	13	Ileal stoma	Bleeding, Resp, Renal, WHD, Redo (10)	Sepsis (peritonitis)	39
M	44	C	22	Duodenal anastomosis	Bleeding, Resp, Renal, HyDecomp, Peritonitis	Sepsis (peritonitis)	51

Resp: respiratory complication, AnaLeak: anastomotic leakage, Redo: redo surgery (*n* = amount of procedures), WHD: wound healing disorder, HRS: hepato-renal syndrome, HyDecomp: hydropic decompensation.

## Supplementary Materials

The following are available online at https://www.mdpi.com/2079-7737/9/11/349/s1, Table S1: Univariate analysis; Table S2: Multivariate analysis; Table S3: Indications for surgery.
