# Supplementary material for: Risk Factors for Postoperative Morbidity and Mortality after Small Bowel Surgery in Patients with Cirrhotic Liver Disease—A Retrospective Analysis of 76 Cases in a Tertiary Center
Source: Biology (Basel). 2020 Oct 22;9(11):349. doi: 10.3390/biology9110349 (PMC7690599; doi:10.3390/biology9110349)
Supplement: Supplementary file 1 [file biology-09-00349-s001.zip › Supplemental Data Tab.1.pdf]

Supplemental data: table S1: Univariate analysis					
	**Hospital Mortality**	**30-Day Mortality**	**D/C ≥ II**	**D/C ≥ IIIB**	**Bleeding requiring transfusion**
Sex	ns	ns	ns	ns	0.001
Age	ns	ns	ns	ns	ns
Age group	ns	ns	ns	ns	ns
Creatinine (mg/dl)	0.036	ns	ns	ns	ns
Total bilirubin (mg/dl)	ns	ns	ns	ns	ns
Bilirubin groups	ns	ns	ns	ns	ns
INR (International normalized Ratio)	0.002	ns	ns	ns	ns
INR group	0.006	ns	ns	ns	ns
MELD score > 9	0.006	ns	ns	ns	ns
MELD score >14	0.001	ns	ns	ns	ns
MELD score > 17	0.007	ns	ns	ns	ns
Model for end-stage liver disease	<0.001	ns	ns	ns	ns
Leucocytes (G/L)	ns	ns	ns	ns	ns
Leucocytosis/penia <3.6 >10.5 (G/L)	ns	ns	ns	ns	ns
Thrombocytes (G/L)	ns	ns	ns	ns	ns
Thrombocytopenia <100 (G/L)	0.036	ns	ns	ns	ns
Ascites group	0.022	ns	ns	ns	ns
No/mild ascites	ns	ns	ns	ns	ns
Moderate ascites	ns	ns	ns	ns	ns
Severe/refractory ascites	0.007	ns	ns	ns	ns
Encephalopathy	0.021	ns	ns	ns	ns
CTP group	0.027	ns	ns	ns	ns
Portal hypertension	0.005	ns	ns	ns	ns
Splenomegaly	ns	ns	ns	ns	ns
Collaterals	ns	ns	ns	ns	ns
Varix	0.032	ns	ns	ns	ns
Hepatocellular carcinoma	ns	ns	ns	ns	ns
Alcoholic	ns	ns	ns	ns	sn
Viral (HBV and/or HCV)	ns	ns	ns	ns	ns
Cryptogenic	ns	ns	ns	ns	ns
Pre-existing metabolic conditions / diabetes	ns	ns	ns	ns	ns
Pre-existing cardiac conditions	ns	ns	ns	ns	ns
Pre-existing renal conditions	0.041	ns	ns	ns	ns
Pre-existing respiratory conditions	ns	ns	ns	ns	ns
Pre-existing neurological conditions	ns	ns	ns	0.005	ns
ASA classification	0.014	ns	ns	0.029	ns
Elective vs. emergency	0.026	ns	ns	ns	ns
Anastomosis 1st surgery	ns	ns	0.042	ns	ns
Additional surgery (other than small bowl)	ns	ns	0.061	ns	ns

ns non-significant (p value ≥ 0.05), *D/C* Dindo/Clavien grade, *Resp* respiratory, *Hydrop decomp* Hydropic decompensation, *CTP* Child Turcotte Pugh, *HBV* hepatitis B virus, *HCV* hepatitis C virus, *ASA* American society of Anesthesiologists,

Supplemental data: table S1: Univariate analysis

	Resp	Renal	Wound healing disorder	Hydrop decomp	Anastomotic leakage	Redo procedure
Sex	ns	ns	ns	ns	ns	ns
Age	ns	ns	ns	ns	ns	ns
Age group	ns	ns	ns	ns	ns	ns
Creatinine (mg/dl)	ns	ns	ns	ns	ns	ns
Total bilirubin (mg/dl)	ns	ns	ns	ns	ns	ns
Bilirubin groups	ns	ns	ns	ns	ns	ns
INR (International normalized Ratio)	ns	0.04	ns	0.042	ns	ns
INR group	ns	0.015	ns	ns	ns	ns
MELD score > 9	ns	ns	ns	ns	ns	ns
MELD score >14	ns	0.047	ns	ns	ns	ns
MELD score > 17	ns	ns	ns	ns	ns	ns
Model for end-stage liver disease	ns	0.042	ns	ns	ns	ns
Leucocytes (G/L)	ns	ns	ns	ns	ns	ns
Leucocytosis/penia <3.6 >10.5 (G/L)	0.036	ns	ns	0.041	ns	ns
Thrombocytes (G/L)	ns	ns	ns	ns	ns	ns
Thrombocytopenia <100 (G/L)	0.01	0.001	ns	0.013	ns	ns
Ascites group	ns	0.045	ns	<0.001	ns	ns
No/mild ascites	0.043	0.044	ns	0.001	0.03	ns
Moderate ascites	ns	ns	0.049	ns	0.001	ns
Severe/refractory ascites	ns	ns	ns	0.01	ns	ns
Encephalopathy	ns	0.001	ns	0.032	ns	ns
CTP group	ns	ns	ns	<0.001	ns	ns
Portal hypertension	ns	0.035	ns	0.003	ns	ns
Splenomegaly	ns	ns	ns	ns	ns	ns
Collaterals	ns	ns	ns	0.005	ns	ns
Varix	ns	0.035	ns	0.002	ns	ns
Hepatocellular carcinoma	0.01	0.038	ns	ns	ns	ns
Alcoholic	ns	ns	ns	ns	ns	ns
Viral (HBV and/or HCV)	ns	ns	ns	ns	ns	ns
Cryptogenic	ns	ns	ns	ns	ns	ns
Pre-existing metabolic conditions / diabetes	ns	ns	ns	ns	ns	ns
Pre-existing cardiac conditions	ns	ns	ns	ns	ns	ns
Pre-existing renal conditions	ns	ns	ns	ns	ns	ns
Pre-existing respiratory conditions	ns	ns	ns	ns	ns	ns
Pre-existing neurological conditions	ns	ns	ns	ns	ns	ns
ASA classification	0.043	0.021	ns	ns	ns	0.042
Elective vs. emergency	0.046	0.021	ns	ns	ns	ns
Anastomosis 1st surgery	ns	ns	ns	ns	ns	ns
Additional surgery (other than small bowl)	ns	ns	ns	0.046	ns	ns

ns non-significant (p value ≥ 0.05), *D/C* Dindo/Clavien grade, *Resp* respiratory, *Hydrop decomp* Hydropic decompensation, *CTP* Child Turcotte Pugh, *HBV* hepatitis B virus, *HCV* hepatitis C virus, *ASA* American society of Anesthesiologists,
