# Supplementary material for: Risk Factors for Postoperative Morbidity and Mortality after Small Bowel Surgery in Patients with Cirrhotic Liver Disease—A Retrospective Analysis of 76 Cases in a Tertiary Center
Source: Biology (Basel). 2020 Oct 22;9(11):349. doi: 10.3390/biology9110349 (PMC7690599; doi:10.3390/biology9110349)
Supplement: Supplementary file 1 [file biology-09-00349-s001.zip › Supplemental Data Tab.2.pdf]

Supplemental data: table S2: Multivariate analysis

	Hospital mortality	OR	D/C ≥ II	OR	D/C ≥ III B	OR	Bleeding requiring transfusion	OR
Model for end-stage liver disease	0.006	1.265	ns	ns	ns	ns	ns	ns
Portal hypertension	0.053	5.782	ns	ns	ns	ns	0.015	4.137
Anastomosis 1st surgery	ns	ns	0.048	5.555	ns	ns	ns	ns
Additional surgery (other than small bowel)	ns	ns	0.064	5.309	ns	ns	ns	ns
Thrombocytopenia <100 (G/L)	ns	ns	ns	ns	0.06	4.964	ns	ns
Pre-existing neurological conditions	ns	ns	ns	ns	0.007	5.579	ns	ns
Sex	ns	ns	ns	ns	ns	ns	0.001	17.845
Elective vs. emergency	ns	ns	ns	ns	ns	ns	0.04	4.662
Leucocytosis/penia <3.6 or >10.5 (G/L)	ns	ns	ns	ns	ns	ns	ns	ns
CTP group	ns	ns	ns	ns	ns	ns	ns	ns
Moderate ascites	ns	ns	ns	ns	ns	ns	ns	ns
Ascites group	ns	ns	ns	ns	ns	ns	ns	ns
ASA score	ns	ns	ns	ns	ns	ns	ns	ns

ns non-significant, *OR* Odds' ratio, *CTP* Child Turcotte Pugh, *ASA* American society of Anesthesiologists, *D/C* Dindo-Clavien grade

Supplemental data: table S2: Multivariate analysis

	Respiratory complication	OR	Renal complication	OR	Hydropic decompensation	OR	Anastomotic leakage	OR
Model for end-stage liver disease	ns	ns	ns	ns	ns	ns	ns	ns
Portal hypertension	ns	ns	ns	ns	0.032	3.424	ns	ns
Anastomosis 1st surgery	ns	ns	ns	ns	ns	ns	ns	ns
Additional surgery (other than small bowl	0.022	8.819	ns	ns	ns	ns	ns	ns
Thrombocytopenia <100 (G/L)	0.01	10.136	0.003	13.16	ns	ns	ns	ns
Pre-existing neurological conditions	ns	ns	ns	ns	ns	ns	ns	ns
Sex	ns	ns	ns	ns	ns	ns	ns	ns
Elective vs. emergency	ns	ns	ns	ns	ns	ns	ns	ns
Leucocytosis/penia <3.6 or >10.5 (G/L)	0.016	2.125	ns	ns	ns	ns	ns	ns
CTP group	ns	ns	ns	ns	0.005	3.727	ns	ns
Moderate ascites	ns	ns	ns	ns	ns	ns	0.004	11.900
Ascites group	ns	ns	0.056	2.666	ns	ns	ns	ns
ASA score	ns	ns	0.056	14.849	ns	ns	ns	ns

ns non-significant, *OR* Odds' ratio, *CTP* Child Turcotte Pugh, *ASA* American society of Anesthesiologists, *D/C* Dindo-Clavien grade
