# Supplementary material for: Risk Factors for Postoperative Morbidity and Mortality after Small Bowel Surgery in Patients with Cirrhotic Liver Disease—A Retrospective Analysis of 76 Cases in a Tertiary Center
Source: Biology (Basel). 2020 Oct 22;9(11):349. doi: 10.3390/biology9110349 (PMC7690599; doi:10.3390/biology9110349)
Supplement: Supplementary file 1 [file biology-09-00349-s001.zip › Supplemental Data Tab.3.pdf]

Supplemental data table S3: Indication for surgery

Indication for surgery	Total (n=76)
Perforation	15 (19.7%)
Tumor	15 (19.7%)
Ischemia	8 (10.5%)
Stoma relocation	14 (18.4%)
Obstructive small bowel	4 (5.3%)
Diverticular disease	3 (3.9%)
Perforation caused by perforated cholecystitis	1 (1.3%)
Ostomy stenosis	2 (2.6%)
Inflammatory bowel disease	2 (2.6%)
Intestinal bleeding	3 (3.9%)
Other	9 (11.8%)
